# Supplementary material for: Morality, self-control, age, type of offence and sentence length as predictors of psychopathy amongst female incarcerated offenders in South Africa
Source: PLoS One. 2024 Mar 28;19(3):e0299847. doi: 10.1371/journal.pone.0299847 (PMC10977693; doi:10.1371/journal.pone.0299847)
Supplement: Attachment — Submitted filename: Feedback_Table_to_Editors_and_Reviewers_2023_11_24.docx [file pone.0299847.s002.docx]

**Feedback Table to Editors and Reviewers**

| **Recommendations by Editor** | **Feedback from Authors** |
| --- | --- |
| Please ensure that your manuscript meets PLOS ONE's style requirements, including those for file naming. | Thank you for making us aware of this. We changed the manner in which the files are named. |
| Please provide additional information regarding the considerations made for the prisoners included in this study. For instance, please discuss whether participants were able to opt out of the study and whether individuals who did not participate receive the same treatment offered to participants. | Thank you for making us aware of the lack of information regarding this in the manuscript. We elaborated on these points under the heading “Ethical Considerations”. We hope these changes address what you requested. |
| Consider depositing your raw data in a repository to ensure your work is read, appreciated and cited by the largest possible audience. You’ll also earn an Accessible Data icon on your published paper if you deposit your data in any participating repository. | Thank you for this recommendation. We considered this recommendation and uploaded a minimal data set to Figshare. <https://doi.org/10.6084/m9.figshare.24631407> |
| In your Data Availability statement, you have not specified where the minimal data set underlying the results described in your manuscript can be found. PLOS defines a study's minimal data set as the underlying data used to reach the conclusions drawn in the manuscript and any additional data required to replicate the reported study findings in their entirety. All PLOS journals require that the minimal data set be made fully available. | Thank you for this recommendation. We considered this recommendation and uploaded a minimal data set to Figshare. <https://doi.org/10.6084/m9.figshare.24631407> |
| Please include your full ethics statement in the ‘Methods’ section of your manuscript file. In your statement, please include the full name of the IRB or ethics committee who approved or waived your study, as well as whether or not you obtained informed written or verbal consent. If consent was waived for your study, please include this information in your statement as well. | Thank you for this request. This study obtained ethical clearance from the General and Human Research Ethics Committee at the University of the Free State with ethical clearance number: UFS-HSD2019/0369/1308. Further approval to conduct this study was obtained from the Department of Correctional Services, South Africa. This information has been added under the heading “Data Collection Procedure”. Additional information was also added under the heading “Ethical considerations”. |
| Please include captions for your Supporting Information files at the end of your manuscript, and update any in-text citations to match accordingly. | A caption has been included at the end of the manuscript for the minimal data set. Please advise if other captions are needed. The tables are included in the text. Thus, it is unclear whether captions are needed for them. |
| **Recommendations by Reviewer** | **Feedback from Authors** |
| The study by Oliveira et al, examined psychopathic traits of female incarcerated offenders in South Africa. Using a quantitative and correlational approach, 139 female from South African correctional centers were analyzed. The research uncovers specific predictor variables, including impulsivity, simple tasks, risk-seeking behavior, and self-centeredness, significantly explaining both primary and secondary psychopathy. The findings are intriguing. However, I recommend a comprehensive restructuring of the paper before publication. A reorganization could enhance the clarity, coherence, and impact of the research, ensuring a more robust presentation of the valuable insights gained from the study. | Thank you for reviewing our manuscript and for the valuable feedback and recommendations. We have attempted to reorganize the manuscript and to address all the recommendations made. |
| Overall, the introduction is excessively long, particularly regarding the decrease in the offender population. With 10 pages dedicated to the introduction, it becomes challenging to grasp the study's significance, objectives, and scientific advancements. Simplifying this section is crucial to enhance clarity and make the content more accessible to readers. | Thank you for this feedback. We attempted to be more concise with regards to the introduction section and attempted to condense the discussion in that section. The introduction now spans over 5 pages instead of 10 pages. |
| The following phrase is quite similar to the original text. I recommend a more direct and concise approach to rewriting the paragraph. “According to Levenson [17], psychopathy should not be viewed as a disorder characterised by a deficiency in neurological systems mediated by one’s own relative anxiety or harm avoidance but should be conceptualised as a pattern of intrinsically antisocial behaviour that is based on judgements relating to the relative importance of one’s own wishes and the rights and well-being of others. Levenson”. | Thank you for picking this up. The sentence and paragraph was reformulated to address this concern and for the discussion here to be more concise. |
| In the methods, the authors mention that “The questionnaires took one to one and a half hours to complete depending on the literacy level of the offenders”. Could you clarify if any evaluator instructed individuals to read the questionnaire or assisted them in this process? | Thank you for raising this point. Additional information was added to address this question and we hope that the added information answers the question raised. |
| The authors mentioned that “The LSRP focuses on two distinct dimensions of psychopathy, namely primary psychopathy and secondary psychopathy:” What does the definition of primary and secondary psychopathology mean from a psychiatric perspective? It is difficult to translate this concept into the domain of modern psychiatry, especially with diagnoses primarily conducted through DSM-V. Could you include an explanation of why you employed this definition? Could you also explain if primary and secondary psychopathology are related to mental health diagnoses such as Major Depressive Disorder or Anxiety Disorders? | Thank you for these questions. A sentence was added indicating that “*Primary psychopathy and secondary psychopathy are often used to describe different subtypes or dimensions within the broader construct of psychopathy*”. Based on Levenson’s definition, “psychopathy should not be seen as a disorder but rather as a pattern of intrinsically antisocial behaviour that is based on judgements relating to the relative importance of one’s own wishes and the rights and well-being of others”. The definition of Levenson was used due the LSRP being used in this study. The LSRP was developed for research purposes and not to be used as a diagnostic tool which appropriately aligns with the aim of the study. |
| The results section is overly extensive. It is difficult to read and to follow the main point of the study. I recommend a complete rewrite for conciseness. Additionally, consider using subtitles related to the findings, not just the authors' analysis. | Thank you for this comment. Two additional headings were included regarding the regression findings. The correlation findings were discussed more concisely. Please see whether you are more comfortable with this more concise discussion. We believe that the discussion of the regression findings is concise and straightforward enough. |
| Tables 4 and 5 are challenging to follow. I recommend presenting these results in a different format for better clarity. | Thank you for this comment. We would prefer to keep the tables as is as the tables do clearly present the findings of the hierarchical regression analysis. Additionally, the findings presented in the tables are explained and discussed in the text below the tables to clarify the findings presented in the tables. |
