# Supplementary material for: Morality, self-control, age, type of offence and sentence length as predictors of psychopathy amongst female incarcerated offenders in South Africa
Source: PLoS One. 2024 Mar 28;19(3):e0299847. doi: 10.1371/journal.pone.0299847 (PMC10977693; doi:10.1371/journal.pone.0299847)
Supplement: Attachment — Submitted filename: Feedback_Table_to_Editors_and_Reviewers_2024_01_24.docx [file pone.0299847.s003.docx]

**Feedback Table to Editors and Reviewers**

| **Recommendations by Reviewer** | **Feedback from Authors** |
| --- | --- |
| Oliveira and colleagues made a substantial improvement in the study. However, there are still some aspects that need further refinement. | Thank you for your valuable recommendations as they assisted in improving the quality of the manuscript. |
| The introduction is too long with five pages. Try to make it shorter and more to the point, presenting the main ideas clearly and concisely. | The authors worked on shortening the introduction section and being more concise regarding the main ideas shared. |
| I suggest incorporating the Purpose of the study and the Research aim and questions in the introduction. | Thank you for this recommendation. The authors incorporated the purpose of the study and the research aim and research questions in the introduction section. |
| For Tables 4 and 5, I recommend replacing the summarizing variables (i.e., “[A + TO + SL]”) with what they represent. For example, [A + TO + SL] = Demographics, [ROC + D + C] = MMS, and [I + ST + RS + SC + T] = SCSM. This way, it’s clear to the reader why those variables are grouped the way they are. | Thank you for this recommendation. The authors made the changes to tables 4 and 5 as requested. |
